# How I treat newly diagnosed acute lymphoblastic leukemia

**DOI:** 10.46989/001c.117026

**Published:** 2024-05-09

**Authors:** Giebel Sebastian

**Affiliations:** 1 Bone Marrow Transplantation and Onco-Hematology Maria Sklodowska-Curie National Research Institute of Oncology Gliwice Branch

**Keywords:** acute lymphoblastic leukemia, induction, minimal residual disease, tyrosine kinase inhibitors, blinatumomab, inotuzumab ozogamycin

## Abstract

Treatment algorithms differ for adult patients with Philadelphia-negative (Ph-) and Philadelphia-positive (Ph+) acute lymphoblastic leukemia (ALL). For Ph- ALL intensive induction-consolidation chemotherapy using “pediatric-inspired” protocols is a standard of care. Allogeneic hematopoietic cell transplantation (allo-HCT) from either an HLA-matched sibling, unrelated or haploidentical donor should be considered for patients with high estimated risk of relapse. Inadequate response at the level of measurable residual disease (MRD) is the strongest adverse prognostic factor. Patients with B-ALL and detectable MRD should be treated with blinatumomab. In the future, the use of blinatumomab and/or inotuzumab ozogamycin in addition to first-line chemotherapy may become a new standard of care reducing the role of allo-HCT. For patients with Ph+ ALL, tyrosine kinase inhibitors (TKI) are the most important components of treatment protocols, while the intensity of chemotherapy may be reduced. Allo-HCT is recommended for all patients treated with imatinib along with low-intensity chemotherapy. Results of phase-II studies using front-line dasatinib or ponatinib in sequence or in combination with blinatumomab are very promising. Such a strategy may allow the avoidance of systemic chemotherapy. The future role of allo-HCT in this context appears uncertain.

## Introduction

Acute lymphoblastic leukemia (ALL) is a heterogeneous disease. According to the International Consensus Classification, more than 30 genetically defined subtypes of precursor lymphoid neoplasms may be distinguished and split into precursor B-cell neoplasms (B-cell acute lymphoblastic leukemia/lymphoma, B-ALL/LBL) and precursor T-cell neoplasms (T-cell acute lymphoblastic leukemia/lymphoma, T-ALL/LBL).^1^ At present, however, from the clinical point of view, only one subtype, i.e., Philadelphia chromosome-positive (Ph+) ALL, characterized by the presence of a t(9;22)(q34.1;q11.2) and corresponding *BCR-ABL1* gene rearrangement requires particular treatment, while for the remaining ones (Philadelphia-negative, Ph- ALL) the first-line therapy is relatively consistent.

The treatment of adults with ALL traditionally consists of polychemotherapy. It includes several phases: a pre-treatment phase, mainly using glucocorticoids, followed by an induction phase, aimed at achieving complete remission (CR), and a consolidation phase to perpetuate the state of CR. Follow-up is either maintenance therapy or an allogeneic hematopoietic cell transplantation (allo-HCT), which is recommended for patients with a high estimated risk of disease recurrence.

In the 21st century, Ph+ and Ph- ALL treatment algorithms evolved differently. With the introduction of tyrosine kinase inhibitors (TKI), the treatment results for Ph+ ALL markedly improved. TKI became a backbone, while the intensity of chemotherapy tended to decrease. In contrast, for Ph-, there was a trend to escalate the intensity of chemotherapy following the pediatric experience. The use of so-called pediatric-inspired regimens (PIR) became a common practice, especially among adolescents and young adults (AYA). However, the definition of PIR and the upper age limit for AYA have not been well defined.

## First-line treatment of Ph- ALL: current status

For patients with Ph- ALL, PIR is a standard of care. A meta-analysis of 25 studies comparing the results of pediatric protocols with those originally designed for adults showed an approximately 20% overall survival advantage at 5 years in favor of the pediatric ones in AYA.^2^ Direct implementation of pediatric protocols may be considered for patients up to 40 years old. The treatment intensity should be tapered for individuals between 40 and 55 years old. For those aged 55 years or more, the therapy should be personalized and adjusted to the performance status and comorbidities.

Details of the first-line treatment protocols differ among countries and study groups. The pre-treatment phase is usually based on glucocorticosteroids, mainly dexamethasone +/- cyclophosphamide (CP), administered for 5-7 days. After the initial reduction of the tumor burden, induction therapy is initiated, usually consisting of anthracyclines, vincristine (VCR), dexamethasone, and asparaginase. It may be followed by a second course, including CP, cytosine arabinoside (AraC), mercaptopurine, and methotrexate (MTX). CR is achieved in approximately 90% of patients.^3^ It is defined as bone marrow blasts < 5%, absence of blasts in the peripheral blood, absence of extramedullary disease, absolute neutrophil count > 1x10^9^/L, platelet count > 100x10^9^/L, and independence of red blood cell transfusions.^4^ The reasons for treatment failure include primary resistance or early death due to treatment complications, both affecting approximately 5% of patients.^3^ The consolidation phase includes alternating courses of chemotherapy, consisting of high doses (HD) of MTX, HD-AraC, CP, etoposide, asparaginase, and dexamethasone. The treatment algorithm may include a re-induction phase, i.e., a repeated course of the drugs used during initial induction. Repeated administration of high doses of polyethylene glycol (PEG)-asparaginase (2000 IU/m^2^) is a feature of PIR.^5^ Although the drug appears critical to increasing the overall treatment efficacy, it is associated with a substantial risk of severe complications, including thrombotic events, hepatotoxicity, and pancreatitis.^6^ The incidence of liver toxicity is higher in adults than in children.^7^ While the induction phase usually lasts 4-8 weeks, the consolidation phase takes approximately 6 months. Either maintenance or allo-HCT follows the consolidation phase. Maintenance lasts up to 2 years and includes mercaptopurine, MTX, vincristine, and glucocorticosteroids.

In parallel to systemic chemotherapy, intrathecal use of cytostatics, usually MTX, AraC, and dexamethasone, is obligatory to prevent relapse in the central nervous system (CNS). The same drugs are used to treat initial CNS involvement, if present. In addition, HD-MTX (usually 3 g/m^2^) administered during consolidation penetrates the blood-brain barrier and contributes to protection from CNS recurrence.^8^

Allo-HCT effectively prevents relapse in ALL adults, combining the conditioning regimen’s anti-leukemic activity and beneficial graft-versus-leukemia reaction mediated by donor-derived immune cells. Unfortunately, it is associated with a significant risk of non-relapse mortality (NRM) affecting 15-22% of patients, depending on the donor type.^9^ Therefore, Ph- ALL allo-HCT in the first CR is considered only for patients with a high estimated risk of relapse. Precise indications vary among study groups.^10^ The most commonly accepted high-risk feature is an inadequate response to induction, detected as measurable residual disease (MRD) or MRD persistence/recurrence after consolidation. MRD can be evaluated by real-time quantitative polymerase chain reaction (RQ-PCR) to detect specific Ig/TCR gene rearrangements, or by multiparametric flow cytometry to detect leukemia-specific phenotypes.^11^ RQ-PCR is more expensive and time-consuming but associated with higher sensitivity (10^-5^), while flow cytometry is fast but less sensitive (10^-4^).^12,13^ The negative prognostic value of detectable MRD has been well-documented using both methods.^14–18^ The sensitivity level may further be increased by using digital droplet PCR or next-generation sequencing.^19^ The latter allows for the detection of ALL subclones. However, these methods have not been sufficiently standardized in ALL and, therefore, cannot be recommended for routine evaluation of MRD. Among other high-risk factors, most study groups consider high initial leukocyte count and adverse genetic subtypes, particularly KMT2A rearranged ALL.^10^

In recent years, immunotherapy appears increasingly important in addition to chemotherapy for adults with Ph- ALL. As demonstrated by the French study group, introducing an anti-CD20 antibody, rituximab, to all treatment phases for patients with CD20-positive ALL contributes to improved event-free survival (EFS) and reduced incidence of relapse.^20^ Such an effect could not be demonstrated by the UK study group that included all patients regardless of CD20 expression.^21^ In the latter study, rituximab was restricted to the induction phase. Although rituximab is not approved for treating ALL, several study groups included it in their treatment protocols.

Data from a retrospective propensity-score analysis by the MD Anderson Cancer Center (MDACC, Houston, USA) suggest improved results with ofatumumab, a fully human anti-CD20 monoclonal antibody, in combination with a hyper-CVAD (CP, VCR, doxorubicin, dexamethasone) protocol when compared to rituximab + hyper-CVAD. However, the differences in EFS and overall survival (OS) did not reach statistical significance.^22^

Blinatumomab, a bispecific anti-CD19/anti-CD3 T-cell enhancer, has been approved for treating patients in CR with MRD levels>10^-3^ in the bone marrow. As shown by the results of the phase II study, it may allow for the eradication of MRD in 78% of patients in the first or subsequent CR.^23^ It may be considered a bridge to allow HCT. However, the need for transplantation after blinatumomab remains controversial.

**Fig. 1.**
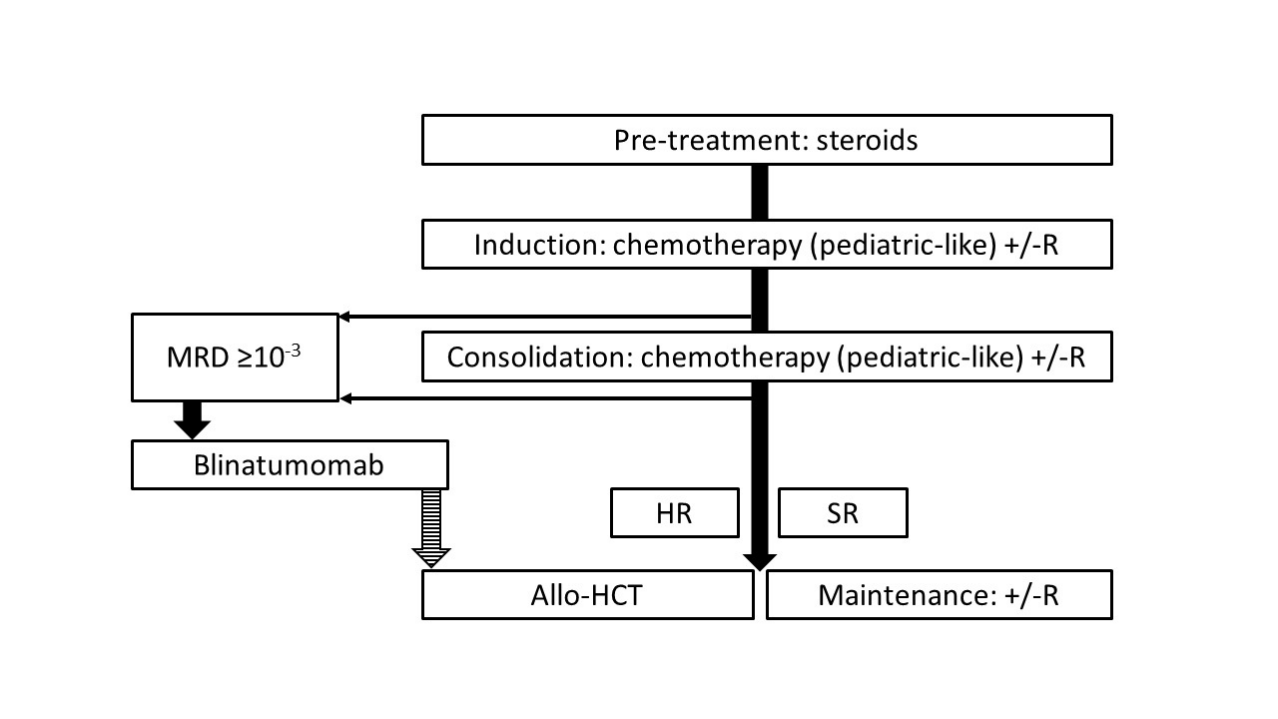
How do I treat newly diagnosed Ph-negative ALL in 2023? R, rituximab; MRD, measurable residual disease; SR, standard risk; HR, high risk; allo-HCT, allogeneic hematopoietic cell transplantation

The use of modern PIR, incorporating all approved and available drugs, offers a chance of long-term survival to 50-70% of adults with Ph- ALL.^24–34^ Relapse and transplant-related mortality are the most frequent causes of treatment failure. The outcomes are inferior in older patients due to higher frequency of adverse molecular subtypes, poor tolerance of intensive chemotherapy and ineligibility for myeloablative allo-HCT.^35^ The current treatment algorithm for adults with Ph- ALL is shown in **Fig. 1**.

## First-line treatment of Ph- ALL: perspectives

While in the first 2 decades of the 21^st^ century the intensity of chemotherapy for patients with Ph- ALL tended to increase, this trend may reverse in view of results of modern studies incorporating immunotherapy to the first-line treatment. A series of phase-2 studies demonstrated feasibility of administration of either binatumomab, inotuzumab ozogamicin (InO, immunoconjugate of ani-CD22 monoclonal antibody and calicheamicin) or both, in combination or in sequence, with standard chemotherapy.^36–39^

In the GIMEMA LAL2317 trial, 2 courses of blinatumomab were administered, in addition to the standard induction-consolidation.^36^ Among 146 patients aged 18-65 years, the probabilities of OS and disease-free survival (DFS) at 12 months were 84% and 72%, respectively. In the French QUEST study, high risk patients, i.e. those presenting *KMT2A*-rearrangement, *IKZF1* intragenic deletion, and/or MRD post-induction ≥10^-4^, received up to 5 cycles of blinatumomab during consolidation and maintenance phases, or as a bridge to allo-HCT.^37^ Among 94 evaluable individuals, the OS rate at 2.5 years was 79%, while the probability of DFS was 72%. In a study by Short et al., 45 patients aged 14-59 years were administered 4-7 cycles of blinatumomab +/- InO in addition to hyper-CVAD regimen.^38^ Three-year continuous remission and OS rates were 84% and 85%, respectively. Among those receiving InO, no relapses or NRM were observed at one year follow-up. Finally, in the ECOG-ACRIN E1910 phase-III trial, patients aged 30-70 years old who achieved CR with MRD <10^-4^ after induction and early intensification were randomly assigned to receive either standard chemotherapy or chemotherapy in sequence with 4 blocks of blinatumomab.^39^ The risk of mortality was significantly reduced in the blinatumomab arm (median OS: not reached versus 71.4 months; hazard ratio = 0.42, 95% CI: 0.24 - 0.75; p=0.003). These data strongly indicate that blinatumomab should be considered a standard of care in both MRD-positive and -negative disease status.

The use of immunotherapy in front-line treatment appears particularly attractive for older patients with Ph- B-ALL. Stelljes et al. reported results of the German phase-II trial where 45 patients with a median age of 64 years (range, 56-80 years) received induction based on InO combined with dexamethasone, followed by 2 courses of InO monotherapy, consolidation, reinduction and maintenance.^40^ The CR rate was 100%, while the probabilities of OS and EFS at 2 years were 81% and 73%, respectively. In the EWALL-INO study, 131 patients with a median age of 68 years (55-84) were induced with low doses of chemotherapy (VCR, CP, dexamethasone) including InO, followed by chemotherapy-based consolidation and maintenance.^41^ The OS and DFS rates at 2 years were 54% and 50%, respectively. In another prospective German study, 34 patients aged 65 (56-76) years were treated with induction, including idarubicin, VCR, and dexamethasone, followed by a single course of blinatumomab in sequence with chemotherapy.^42^ OS and DFS rates at 1 year were 84% and 89%, respectively. Overall, the results of the above-cited trials are much better than historical data with chemotherapy only, giving hope for a cure in older patients with Ph- B-ALL.^43^

T-ALL accounts for 20-25% of adult ALL. So far, humoral immunotherapy forms are unavailable for this disease subtype. Conventional chemotherapy remains a backbone, with attempts to personalize treatment by incorporating nelarabine in first-line protocols. In the UKALL14 trial, three doses of nelarabine were administered following the second phase of induction.^44^ This addition had no impact on any of the study outcomes. On the other hand, according to a retrospective analysis by MDACC, the use of nelarabine may contribute to improved outcomes in T-ALL subtypes other than early T-cell precursor ALL.^45^ Further prospective studies are needed to verify this finding.

Ph-like ALL is a disease subtype characterized by a gene expression profile similar to Ph+ ALL in the absence of *BCR-ABL1* fusion gene.^46^ This subtype may be further subclassified according to the type of molecular alterations and affected pathways, and is associated with poor outcomes.^47^ ABL1 class mutations may be targeted by TKIs, like imatinib or dasatinib. JAK2 fusions, EPOR rearrangements, and activating JAK-STAT mutations are potentially sensitive to the JAK2 inhibitor ruxolitinib.^48^ The use of these agents in first-line treatment is under clinical investigation. So far, however, data on adults are scarce. Furthermore, the identification of Ph-like ALL is not routine clinical practice.

**Fig. 2.**
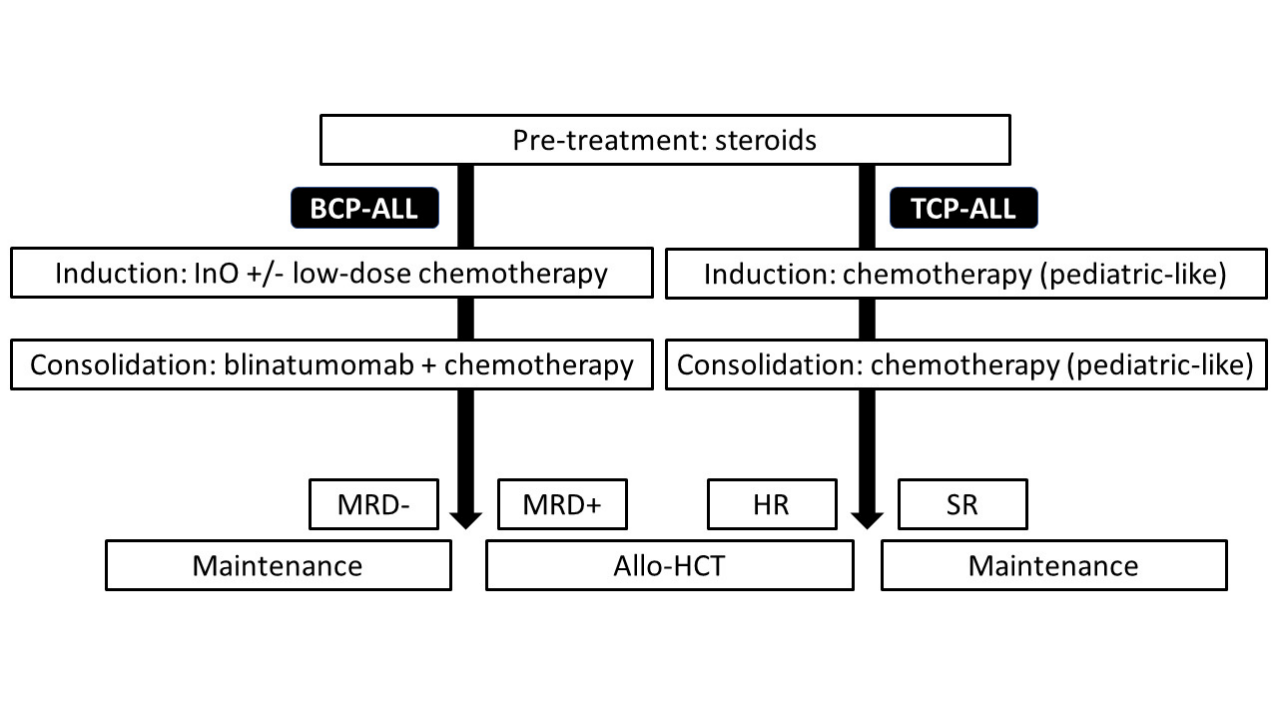
How would I like to treat newly diagnosed Ph-negative ALL? BCP-ALL, B-cell precursor acute lymphoblastic leukemia; TCP-ALL, T-cell precursor acute lymphoblastic leukemia; InO, inotuzumab ozogamycin; MRD, measurable residual disease; SR, standard risk; HR, high risk; allo-HCT, allogeneic hematopoietic cell transplantation

Perspectives for treating adults with Ph- ALL are presented in **Fig. 2**.

## First-line treatment of Ph+ ALL: current status

Ph+ ALL is the most common molecular subtype. Its frequency increases with age, being 5-15% in adolescents, 25-30% in patients aged 25-35 years and more than 35-40% in patients older than 35 years.^49,50^ Historically, Ph+ ALL was associated with a dismal prognosis, and considered a very high-risk subtype. Results improved dramatically with the introduction of TKIs.^51,52^ So far, imatinib is the only TKI approved for use in first-line treatment. The combination of imatinib with either corticosteroids or multi-agent chemotherapy results in 90-100% CR rate.^53–56^ Unfortunately, without subsequent allo-HCT most patients experience relapse. The role of intensity of concomitant chemotherapy was addressed in the GRAAPH-2005 study.^57^ Patients were randomly assigned to receive induction consisting of either imatinib in combination with vincristine and dexamethasone or imatinib + hyper-CVAD. CR rates were 98.5% and 91% (p=0.006), respectively, with increased early mortality observed in the hyper-CVAD arm. The 5-year EFS and OS rates were higher for the “low-intensity” arm, although the differences were not significant. Allo-HCT was intended for all patients. A post-hoc analysis revealed improved outcomes for those having an HLA-matched sibling donor (MSD) or matched unrelated donor (MUD) and actually treated with allo-HCT compared to the remaining individuals. Based on results of that study the use of imatinib in combination with low intensity chemotherapy became a standard of care. It should be accompanied by intrathecal prophylaxis of CNS relapse, as well as high doses of methotrexate in consolidation. Allo-HCT is recommended for all eligible patients. Strict MRD monitoring, based on BCR-ABL1 transcript detection, is obligatory. Persistence or recurrence of MRD may implicate the introduction of a second (dasatinib) or third (ponatinib) generation TKI, optimally after verification of the presence of point mutations within the ABL1 kinase domain. The use of TKI is also recommended as a maintenance after allo-HCT. It may be introduced either prophylactically, regardless of MRD status, or as a pre-emptive approach, in case of MRD-positivity.^58^ Detailed guidelines have been elaborated by the European Society for Blood and Marrow Transplantation (EBMT).^59^

**Fig. 3.**
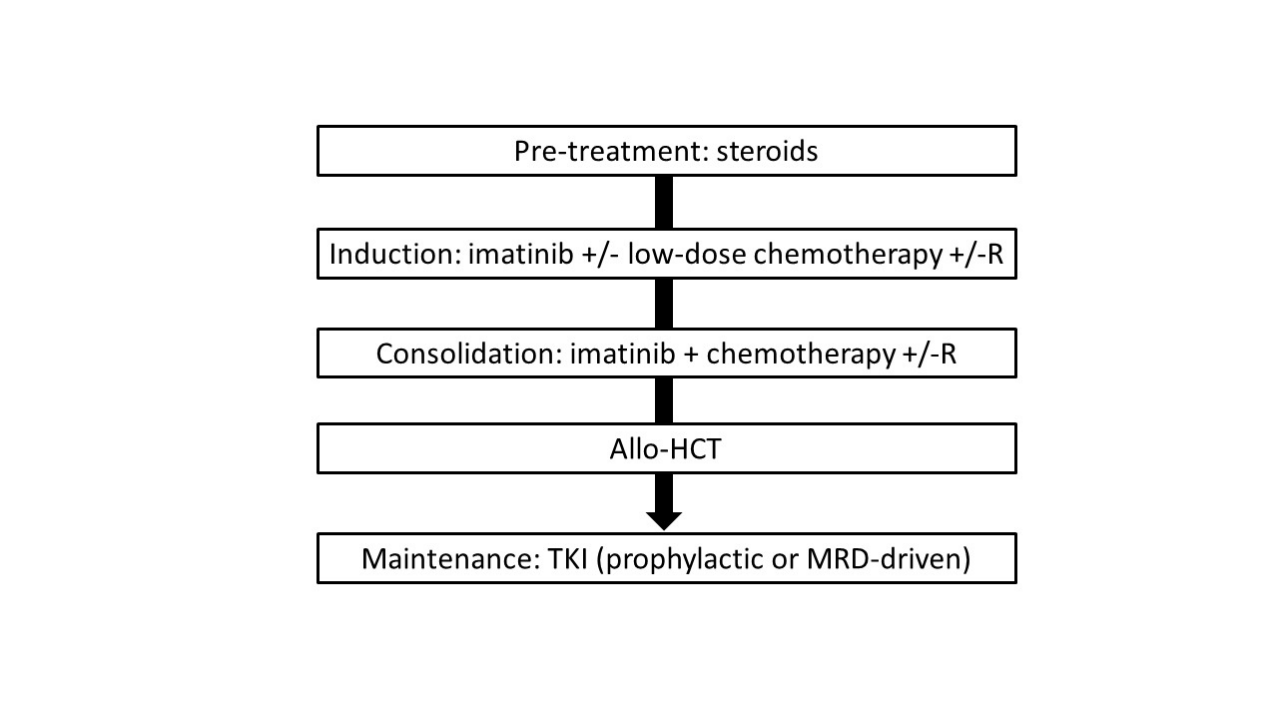
How do I treat newly diagnosed Ph-positive ALL in 2023? R, rituximab; allo-HCT, allogeneic hematopoietic cell transplantation; TKI, tyrosine kinase inhibitor; MRD, measurable residual disease

The current treatment standard for adults with Ph+ ALL is presented in **Fig. 3**.

## First-line treatment of Ph+ ALL: perspectives

The upfront use of second or third generation TKI may contribute to an increased rate of molecular responses. When combined with intensive chemotherapy and/or modern immunotherapy, there is a potential for cure without allo-HCT. The only prospective, randomized trial comparing imatinib and dasatinib administered in parallel to intensive chemotherapy was performed in the pediatric population.^60^ The use of dasatinib was associated with improved EFS (71% versus 49% at 4 years) and OS (88% versus 69% at 4 years), with reduced incidence of relapse, including CNS relapse. Dasatinib is the only TKI that crosses the blood-brain barrier.

In the adult population, only results of phase-II studies are available. The combination of dasatinib with hyper-CVAD led to 96% CR rate and 46% probability of the OS at 5 years, with only 17% of patients treated with allo-HCT.^61^ The outcomes appear even better for ponatinib used in parallel to hyper-CVAD: 98% CR, 67% EFS and 71% OS rates at 5 years, with 30% of patients treated with allo-HCT.^62^ In the older population (>55 years old) dasatinib combined with intermediate dose chemotherapy allowed for a 36% 5-year OS rate, while allo-HCT was applied to 10% of patients.^63^

An Italian group performed a study using dasatinib + dexamethasone in induction, followed by 2-5 cycles of blinatumomab and dasatinib maintenance.^64^ The CR rate was 98%, including 60% molecular responses. EFS and OS rates at 18 months were 88% and 95%, respectively. The treatment was free from systemic chemotherapy except for 38% of patients referred for allo-HCT. Jabbour et al. performed a study combining ponatinib and blinatumomab for up to 5 cycles with subsequent ponatinib maintenance.^65^ Among 40 patients with median age of 57 (20-83) years, 96% achieved CR, while the probability of OS at 2 years was 95%. Only one patient received allo-HCT in CR1.

**Fig. 4.**
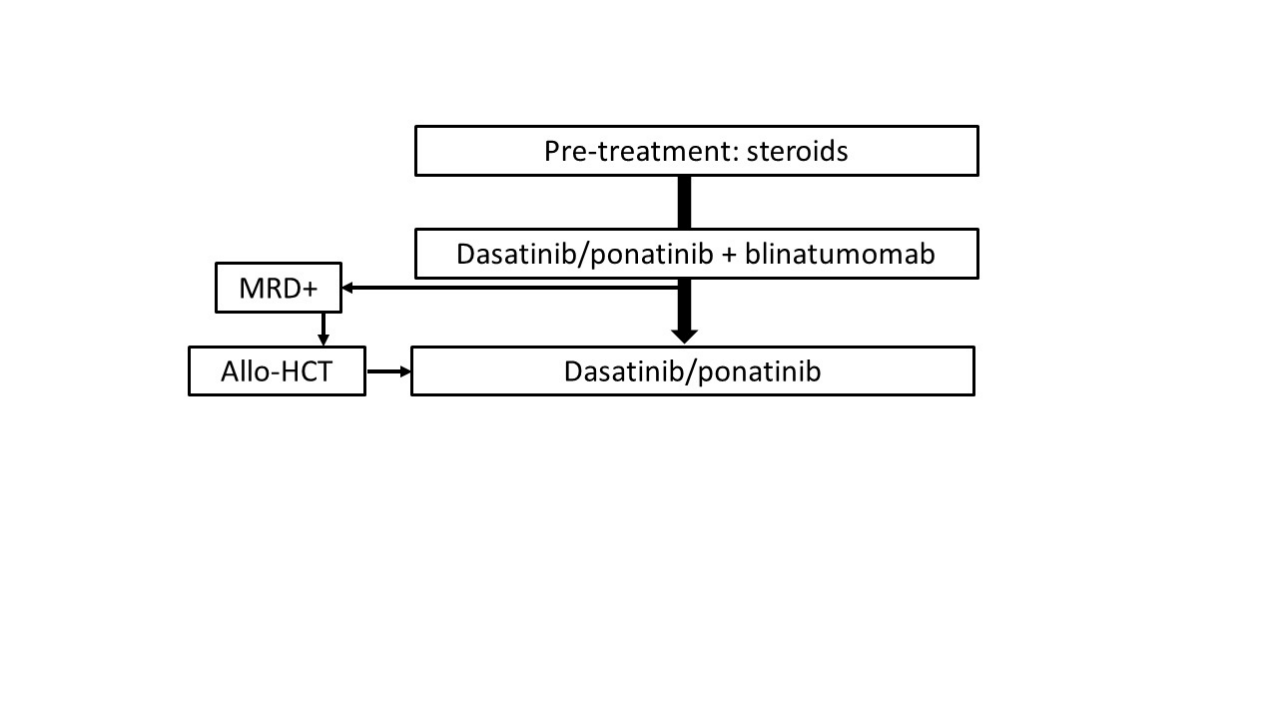
How would I like to treat newly diagnosed Ph-positive ALL? MRD, measurable residual disease; allo-HCT, allogeneic hematopoietic cell transplantation

Taken together, it seems that the use of second or third generation TKI in combination or in sequence with blinatumomab may markedly improve the prognosis of patients with Ph+ ALL, reducing the role of allo-HCT as part of the first-line treatment. However, longer follow-up is needed to draw final conclusions. The perspectives of the treatment of adults with Ph+ ALL are summarized in **Fig. 4**.

## Allo-HCT in first-line treatment

Allo-HCT is currently recommended for high-risk patients with Ph- ALL and all patients with Ph+ ALL treated with upfront imatinib. The choice of potential donors is wide, including MSD, MUD, mismatched unrelated donors (MMUD) and haploidentical relatives. The intensity of conditioning may be adjusted to the recipient’s biological status, which means that almost all patients with indications for allo-HCT may be offered this procedure.

The impact of donor type on the results of allo-HCT in ALL was a subject of several retrospective analyses. In a study by the EBMT Acute Leukemia Working Party, no significant difference could be detected between transplantations from MUD, MMUD and haploidentical donors.^66^ In another study, allo-HCT from haploidentical donors was associated with increased non-relapse-mortality compared to MSD transplants which, however, was counterbalanced by a reduced risk of relapse.^67^ Hence, it seems that in terms of survival, all donor types may be considered equally valuable.

Studies focused on haploidentical transplantations showed better results for unmanipulated grafts with the use of post-transplant cyclophosphamide as a backbone of immunosuppression, compared to regimens based on anti-thymocyte globulin.^68^ Bone marrow, as compared to peripheral blood as a source of stem cells, was associated with improved OS and leukemia-free survival, mainly as a consequence of reduced risk of GVHD and non-relapse mortality.^69^ Finally, as shown by the EBMT, results of haploidentical transplantations for patients with ALL tend to improve over time.^70^

The outcome of allo-HCT may be affected by the type of conditioning regimen. Results from retrospective analyses indicated that the use of myeloablative total body irradiation (TBI) was associated with a reduced risk of relapse and improved survival, compared to chemotherapy-based regimens.^9,71,72^ These findings were confirmed by a global study on a pediatric population. The outcomes were significantly better for patients treated with TBI at a dose of 12 Gy combined with etoposide, than for those treated with thiotepa and fludarabine combined with either busulfan or treosulfan.^73^ On the other hand, a prospective study in adults indicated non-inferiority of busulfan + cyclophosphamide compared to TBI at a total dose of 9 Gy + cyclophosphamide.^74^ That study, however, was restricted to B-ALL with standard-risk cytogenetics. Therefore, the choice of TBI dose as well as chemotherapy counterpart may play a role. The use of anti-thymocyte globulin for patients with ALL is a matter of controversy, as results from large scale registry-based studies indicate reduced incidence of chronic GVHD but increased risk of relapse in both Ph- and Ph+ ALL^75,76^

## CAR T-cells now and in future

Chimeric antigen receptor (CAR) T-cells are genetically engineered T lymphocytes able to recognize and destroy cancer cells. Two products representing autologous CAR T-cells, tisagenlecleucel and brexucaptagene autoleucel have been approved for the treatment of relapsed/refractory B-ALL.^77,78^ Such therapy could potentially be offered to patients in CR1 with detectable MRD. In this clinical situation, CAR T-cells would be a final treatment, allowing to avoid allo-HCT. Clinical studies exploring this concept are ongoing.

Multiple preclinical and clinical studies are being conducted with the aim to optimize CAR T-cell products in terms of both their efficacy and safety. Among others, this includes simultaneous or sequential targeting of two antigens, as well as the ability to switch-off and switch-on the expression of the CAR.^79^ Similarly, allogeneic CAR T-cells are being engineered, with the ogjective to simplify the logistics of the procedure.^80^ Taking into account its very high research and development potential, it can be hypothesized that, in the future, CAR T-cells will become a predominant treatment tool, and may substitute all other forms of systemic therapy.

## Summary

For adults with Ph- ALL, conventional chemotherapy using PIR remains a standard of care. Allo-HCT from either MSD, MUD, MMUD or haploidentical donors should be considered for patients with high risk of relapse. Patients with B-ALL and detectable MRD should be treated with blinatumomab (**Fig. 1**). In the future, the use of blinatumomab and/or InO in addition to first-line chemotherapy may become a new standard of care reducing the role of allo-HCT (**Fig. 2**).

For patients with Ph+ ALL the use of TKI is a priority. Imatinib in combination with reduced-intensity chemotherapy, followed by allo-HCT is currently recommended (**Fig. 3**). Front-line dasatinib or ponatinib, in sequence or in combination with blinatumomab, is a promising strategy, which may allow to avoid systemic chemotherapy. The future role of allo-HCT in this context appears uncertain (**Fig. 4.

### Competing Interests

Novartis (advisory boards, speakers bureau, honoraria), Gilead (advisory boards, speakers bureau, honoraria), Amgen (advisory boards, speakers bureau, honoraria), Pfizer (advisory boards, speakers bureau, honoraria), Roche (advisory boards, speakers bureau, honoraria), Angelini (advisory boards, speakers bureau, honoraria), Servier (speakers bureau, honoraria).

## References

[ref-308711] Arber D. A., Orazi A., Hasserjian R. P., Borowitz M. J., Calvo K. R., Kvasnicka H. M., Wang S. A., Bagg A., Barbui T., Branford S., Bueso-Ramos C. E., Cortes J. E., Dal Cin P., DiNardo C. D., Dombret H., Duncavage E. J., Ebert B. L., Estey E. H., Facchetti F., Foucar K., Gangat N., Gianelli U., Godley L. A., Gökbuget N., Gotlib J., Hellström-Lindberg E., Hobbs G. S., Hoffman R., Jabbour E. J., Kiladjian J. J., Larson R. A., Le Beau M. M., Loh M. L., Löwenberg B., Macintyre E., Malcovati L., Mullighan C. G., Niemeyer C., Odenike O. M., Ogawa S., Orfao A., Papaemmanuil E., Passamonti F., Porkka K., Pui C. H., Radich J. P., Reiter A., Rozman M., Rudelius M., Savona M. R., Schiffer C. A., Schmitt-Graeff A., Shimamura A., Sierra J., Stock W. A., Stone R. M., Tallman M. S., Thiele J., Tien H. F., Tzankov A., Vannucchi A. M., Vyas P., Wei A. H., Weinberg O. K., Wierzbowska A., Cazzola M., Döhner H., Tefferi A. (2022). International Consensus Classification of Myeloid Neoplasms and Acute Leukemias: integrating morphologic, clinical, and genomic data. Blood.

[ref-308712] Siegel S. E., Stock W., Johnson R. H., Advani A., Muffly L., Douer D., Reed D., Lewis M., Freyer D. R., Shah B., Luger S., Hayes-Lattin B., Jaboin J. J., Coccia P. F., DeAngelo D. J., Seibel N., Bleyer A. (2018). Pediatric-Inspired Treatment Regimens for Adolescents and Young Adults With Philadelphia Chromosome-Negative Acute Lymphoblastic Leukemia: A Review. JAMA Oncol.

[ref-308713] Bassan R., Hoelzer D. (2011). Modern therapy of acute lymphoblastic leukemia. J Clin Oncol.

[ref-308714] Buchmann S., Schrappe M., Baruchel A., Biondi A., Borowitz M., Campbell M., Cario G., Cazzaniga G., Escherich G., Harrison C.J., Heyman M., Hunger S.P., Kiss C., Liu H.C., Locatelli F., Loh M.L., Manabe A., Mann G., Pieters R., Pui C.H., Rives S., Schmiegelow K., Silverman L.B., Stary J., Vora A., Brown P. (2022). Remission, treatment failure, and relapse in pediatric ALL: an international consensus of the Ponte-di-Legno Consortium. Blood.

[ref-308715] Bender C., Maese L., Carter-Febres M., Verma A. (2021). Clinical Utility of Pegaspargase in Children, Adolescents and Young Adult Patients with Acute Lymphoblastic Leukemia: A Review. Blood Lymphat Cancer.

[ref-308716] Piatkowska-Jakubas B., Krawczyk-Kuliś M., Giebel S., Adamczyk-Cioch M., Czyz A., Lech Marańda E., Paluszewska M., Pałynyczko G., Piszcz J., Hołowiecki J., Polish Adult Leukemia Group (2008). Use of L-asparaginase in acute lymphoblastic leukemia: recommendations of the Polish Adult Leukemia Group. Pol Arch Med Wewn.

[ref-308717] Douer D., Gokbuget N., Stock W., Boissel N. (2022). Optimizing use of L-asparaginase-based treatment of adults with acute lymphoblastic leukemia. Blood Rev.

[ref-308718] Giebel S., Krawczyk-Kuliś M., Adamczyk-Cioch M., Czyz A., Lech-Marańda E., Piatkowska-Jakubas B., Paluszewska M., Pałynyczko G., Piszcz J., Hołowiecki J., Polish Adult Leukemia Group (2008). Prophylaxis and therapy of central nervous system involvement in adult acute lymphoblastic leukemia: recommendations of the Polish Adult Leukemia Group. Pol Arch Med Wewn.

[ref-308719] Giebel S., Labopin M., Socié G., Beelen D., Browne P., Volin L., Kyrcz-Krzemien S., Yakoub-Agha I., Aljurf M., Wu D., Michallet M., Arnold R., Mohty M., Nagler A. (2017). Improving results of allogeneic hematopoietic cell transplantation for adults with acute lymphoblastic leukemia in first complete remission: an analysis from the Acute Leukemia Working Party of the European Society for Blood and Marrow Transplantation. Haematologica.

[ref-308720] Giebel S., Marks D. I., Boissel N., Baron F., Chiaretti S., Ciceri F., Cornelissen J. J., Doubek M., Esteve J., Fielding A., Foa R., Gorin N. C., Gökbuget N., Hallböök H., Hoelzer D., Paravichnikova E., Ribera J. M., Savani B., Rijneveld A. W., Schmid C., Wartiovaara-Kautto U., Mohty M., Nagler A., Dombret H. (2019). Hematopoietic stem cell transplantation for adults with Philadelphia chromosome-negative acute lymphoblastic leukemia in first remission: a position statement of the European Working Group for Adult Acute Lymphoblastic Leukemia (EWALL) and the Acute Leukemia Working Party of the European Society for Blood and Marrow Transplantation (EBMT). Bone Marrow Transplant.

[ref-308721] Szczepański T. (2007). Why and how to quantify minimal residual disease in acute lymphoblastic leukemia?. Leukemia.

[ref-308722] van Dongen J.J., van der Velden V.H., Brüggemann M., Orfao A. (2015). Minimal residual disease diagnostics in acute lymphoblastic leukemia: need for sensitive, fast, and standardized technologies. Blood.

[ref-308723] Shaver A. C., Greig B. W., Mosse C. A., Seegmiller A. C. (2015). B-ALL minimal residual disease flow cytometry: an application of a novel method for optimization of a single-tube model. Am J Clin Pathol.

[ref-308724] Holowiecki J., Krawczyk-Kulis M., Giebel S., Jagoda K., Stella-Holowiecka B., Piatkowska-Jakubas B., Paluszewska M., Seferynska I., Lewandowski K., Kielbinski M., Czyz A., Balana-Nowak A., Król M., Skotnicki A. B., Jedrzejczak W. W., Warzocha K., Lange A., Hellmann A. (2008). Status of minimal residual disease after induction predicts outcome in both standard and high-risk Ph-negative adult acute lymphoblastic leukaemia. The Polish Adult Leukemia Group ALL 4-2002 MRD Study. Br J Haematol.

[ref-308725] Bruggemann M., Raff T., Flohr T., Gökbuget N., Nakao M., Droese J., Lüschen S., Pott C., Ritgen M., Scheuring U., Horst H.A., Thiel E., Hoelzer D., Bartram C.R., Kneba M., German Multicenter Study Group for Adult Acute Lymphoblastic Leukemia (2006). Clinical significance of minimal residual disease quantification in adult patients with standard-risk acute lymphoblastic leukemia. Blood.

[ref-308726] Mortuza F. Y., Papaioannou M., Moreira I. M., Coyle L. A., Gameiro P., Gandini D., Prentice H. G., Goldstone A., Hoffbrand A. V., Foroni L. (2002). Minimal residual disease tests provide an independent predictor of clinical outcome in adult acute lymphoblastic leukemia. J Clin Oncol.

[ref-308727] Vidriales M. B., Perez J. J., Lopez-Berges M. C., Gutiérrez N., Ciudad J., Lucio P., Vazquez L., García-Sanz R., del Cañizo M. C., Fernández-Calvo J., Ramos F., Rodríguez M. J., Calmuntia M. J., Porwith A., Orfao A., San-Miguel J. F. (2003). Minimal residual disease in adolescent (older than 14 years) and adult acute lymphoblastic leukemias: early immunophenotypic evaluation has high clinical value. Blood.

[ref-308728] Gokbuget N., Dombret H., Giebel S., Bruggemann M., Doubek M., Foà R., Hoelzer D., Kim C., Martinelli G., Parovichnikova E., Rambaldi A., Ribera J.M., Schoonen M., Stieglmaier J.M., Zugmaier G., Bassan R. (2019). Minimal residual disease level predicts outcome in adults with Ph-negative B-precursor acute lymphoblastic leukemia. Hematology.

[ref-308729] Starza Della I., De Novi L. A., Elia L., Bellomarino V., Beldinanzi M., Soscia R., Cardinali D., Chiaretti S., Guarini A., Foà R. (2023). Optimizing Molecular Minimal Residual Disease Analysis in Adult Acute Lymphoblastic Leukemia. Cancers (Basel).

[ref-308730] Maury S., Chevret S., Thomas X., Heim D., Leguay T., Huguet F., Chevallier P., Hunault M., Boissel N., Escoffre-Barbe M., Hess U., Vey N., Pignon J. M., Braun T., Marolleau J. P., Cahn J. Y., Chalandon Y., Lhéritier V., Beldjord K., Béné M. C., Ifrah N., Dombret H. (2016). Addition of Rituximab in B-Lineage Adult Acute Lymphoblastic Leukemia. N Engl J Med.

[ref-308731] Marks D. I., Kirkwood A. A., Rowntree C. J., Aguiar M., Bailey K. E., Beaton B., Cahalin P., Castleton A. Z., Clifton-Hadley L., Copland M., Goldstone A. H., Kelly R., Lawrie E., Lee S., McMillan A. K., McMullin M. F., Menne T. F., Mitchell R. J., Moorman A. V., Patel B., Patrick P., Smith P., Taussig D., Yallop D., Alapi K. Z., Fielding A. K. (2022). Addition of four doses of rituximab to standard induction chemotherapy in adult patients with precursor B-cell acute lymphoblastic leukaemia (UKALL14): a phase 3, multicentre, randomised controlled trial. Lancet Haematol.

[ref-308732] Sasaki K., Kantarjian H. M., Morita K., Short N. J., Konopleva M., Jain N., Ravandi F., Garcia-Manero G., Wang S., Khoury J. D., Jorgensen J. L., Champlin R. E., Khouri I. F., Kebriaei P., Schroeder H. M., Khouri M., Garris R., Takahashi K., O'Brien S. M., Jabbour E. J. (2021). Hyper-CVAD plus ofatumumab versus hyper-CVAD plus rituximab as frontline therapy in adults with Philadelphia chromosome-negative acute lymphoblastic leukemia: A propensity score analysis. Cancer.

[ref-308733] Gokbuget N., Dombret H., Bonifacio M., Reichle A., Graux C., Faul C., Diedrich H., Topp M. S., Brüggemann M., Horst H. A., Havelange V., Stieglmaier J., Wessels H., Haddad V., Benjamin J. E., Zugmaier G., Nagorsen D., Bargou R. C. (2018). Blinatumomab for minimal residual disease in adults with B-precursor acute lymphoblastic leukemia. Blood.

[ref-308734] Bassan R., Chiaretti S., Della Starza I., Spinelli O., Santoro A., Elia L., De Propris M. S., Scattolin A. M., Paoloni F., Messina M., Audisio E., Marbello L., Borlenghi M., Zappasodi P., Vetro C., Martinelli G., Mattei D., Fracchiolla N., Bocchia M., De Fabritiis P., Bonifacio M., Candoni A., Cassibba V., Di Bartolomeo P., Latte G., Trappolini S., Guarini A., Vitale A., Fazi P., Vignetti M., Rambaldi A., Foà R. (2022). National PEGASPARGASE-modified risk-oriented program for Philadelphia-negative adult acute lymphoblastic leukemia/lymphoblastic lymphoma (PH-ALL/LL). GIMEMA LAL 1913 final results. HemaSphere.

[ref-308735] Gökbuget N., Stelljes M., Viardot A., Nachtkamp K., Steffen B. (2021). First Results of the Risk-Adapted, MRD-Stratified GMALL Trial 08/2013 in 705 Adults with Newly Diagnosed Acute Lymphoblastic Leukemia/Lymphoma (ALL/LBL). Blood.

[ref-308736] Huguet F., Chevret S., Leguay T., Thomas X., Boissel N., Escoffre-Barbe M., Chevallier P., Hunault M., Vey N., Bonmati C., Lepretre S., Marolleau J.P., Pabst T., Rousselot P., Buzyn A., Cahn J.Y., Lhéritier V., Béné M.C., Asnafi V., Delabesse E., Macintyre E., Chalandon Y., Ifrah N., Dombret H. (2018). Intensified Therapy of Acute Lymphoblastic Leukemia in Adults: Report of the Randomized GRAALL-2005 Clinical Trial. J Clin Oncol.

[ref-308737] Rijneveld A. W., van der Holt B., de Weerdt O., Biemond B. J., van de Loosdrecht A. A., van der Wagen L. E., Bellido M., van Gelder M., van der Velden W. J. F. M., Selleslag D., van Lammeren-Venema D., Halkes C. J. M., Fijnheer R., Havelange V., van Sluis G. L., Legdeur M. C., Deeren D., Gadisseur A., Sinnige H. A. M., Breems D. A., Jaspers A., Legrand O., Terpstra W. E., Boersma R. S., Mazure D., Triffet A., Tick L. W., Beel K., Maertens J. A., Beverloo H. B., Bakkus M., Homburg C. H. E., de Haas V., van der Velden V. H. J., Cornelissen J. J. (2022). Clofarabine added to intensive treatment in adult patients with newly diagnosed ALL: the HOVON-100 trial. Blood Adv.

[ref-308738] Sakura T., Hayakawa F., Sugiura I., Murayama T., Imai K., Usui N., Fujisawa S., Yamauchi T., Yujiri T., Kakihana K., Ito Y., Kanamori H., Ueda Y., Miyata Y., Kurokawa M., Asou N., Ohnishi K., Ohtake S., Kobayashi Y., Matsuo K., Kiyoi H., Miyazaki Y., Naoe T. (2018). High-dose methotrexate therapy significantly improved survival of adult acute lymphoblastic leukemia: a phase III study by JALSG. Leukemia.

[ref-308739] Rytting M. E., Jabbour E. J., Jorgensen J. L., Ravandi F., Franklin A. R., Kadia T. M., Pemmaraju N., Daver N. G., Ferrajoli A., Garcia-Manero G., Konopleva M. Y., Borthakur G., Garris R., Wang S., Pierce S., Schroeder K., Kornblau S. M., Thomas D. A., Cortes J. E., O'Brien S. M., Kantarjian H. M. (2016). Final results of a single institution experience with a pediatric-based regimen, the augmented Berlin-Frankfurt-Munster, in adolescents and young adults with acute lymphoblastic leukemia, and comparison to the hyper-CVAD regimen. Am J Hematol.

[ref-308740] Bassan R., Pavoni C., Intermesoli T., Spinelli O., Tosi M., Audisio E., Marmont F., Cattaneo C., Borlenghi E., Cortelazzo S., Cavattoni I., Fumagalli M., Mattei D., Romani C., Cortelezzi A., Fracchiolla N., Ciceri F., Bernardi M., Scattolin A. M., Depaoli L., Masciulli A., Oldani E., Rambaldi A. (2020). Updated risk-oriented strategy for acute lymphoblastic leukemia in adult patients 18-65 years: NILG ALL 10/07. Blood Cancer Journal.

[ref-308741] Toft N., Birgens H., Abrahamsson J., Griškevičius L., Hallböök H., Heyman M., Klausen T. W., Jónsson Ó. G., Palk K., Pruunsild K., Quist-Paulsen P., Vaitkeviciene G., Vettenranta K., Åsberg A., Frandsen T. L., Marquart H. V., Madsen H. O., Norén-Nyström U., Schmiegelow K. (2018). Results of NOPHO ALL2008 treatment for patients aged 1-45 years with acute lymphoblastic leukemia. Leukemia.

[ref-308742] Ribera J. M., Morgades M., Ciudad J., Montesinos P., Esteve J., Genescà E., Barba P., Ribera J., García-Cadenas I., Moreno M. J., Martínez-Carballeira D., Torrent A., Martínez-Sánchez P., Monsalvo S., Gil C., Tormo M., Artola M. T., Cervera M., González-Campos J., Rodríguez C., Bermúdez A., Novo A., Soria B., Coll R., Amigo M. L., López-Martínez A., Fernández-Martín R., Serrano J., Mercadal S., Cladera A., Giménez-Conca A., Peñarrubia M. J., Abella E., Vall-Llovera F., Hernández-Rivas J. M., Garcia-Guiñon A., Bergua J. M., de Rueda B., Sánchez-Sánchez M. J., Serrano A., Calbacho M., Alonso N., Méndez-Sánchez J. Á., García-Boyero R., Olivares M., Barrena S., Zamora L., Granada I., Lhermitte L., Feliu E., Orfao A. (2021). Chemotherapy or allogeneic transplantation in high-risk Philadelphia chromosome-negative adult lymphoblastic leukemia. Blood.

[ref-308743] Hough R., Rowntree C., Goulden N., Mitchell C., Moorman A., Wade R., Vora A. (2016). Efficacy and toxicity of a paediatric protocol in teenagers and young adults with Philadelphia chromosome negative acute lymphoblastic leukaemia: results from UKALL 2003. Br J Haematol.

[ref-308744] Stock W., Luger S. M., Advani A. S., Yin J., Harvey R. C., Mullighan C. G., Willman C. L., Fulton N., Laumann K. M., Malnassy G., Paietta E., Parker E., Geyer S., Mrózek K., Bloomfield C. D., Sanford B., Marcucci G., Liedtke M., Claxton D. F., Foster M. C., Bogart J. A., Grecula J. C., Appelbaum F. R., Erba H., Litzow M. R., Tallman M. S., Stone R. M., Larson R. A. (2019). A pediatric regimen for older adolescents and young adults with acute lymphoblastic leukemia: results of CALGB 10403. Blood.

[ref-308745] Jammal N., Kantarjian H.M., Haddad F., Jabbour E.J. (2022). Management of acute lymphoblastic leukemia in older adults. Clin Adv Hematol Oncol.

[ref-308746] Bassan R., Chiaretti S., Della Starza I., Spinelli O., Santoro A., Elia L., Vitale A., Taherinasab A., Piccini M., Ferrara F., Sica S., Zappasodi P., Borlenghi E., Bocchia M., Califano C., Di Raimondo F., Pane F., Candoni A., Ruggeri M., Audisio E., Lunghi M., Mianulli A.M., Messina M., Marino M.R., Arena V., Rambaldi A., Foà R. (2021). Preliminary results of the GIMEMA LAL2317 sequential chemotherapy-blinatumomab frontline trial for newly diagnosed adult Ph-negative B-lineage ALL patients. HemaSphere.

[ref-308747] Boissel N., Huguet F., Leguay T., Hunault M., Kim R.. (2022). Blinatumomab during Consolidation in High-Risk Philadelphia Chromosome (Ph)-Negative B-Cell Precursor (BCP) Acute Lymphoblastic Leukemia (ALL) Adult Patients: A Two-Cohort Comparison within the Graall-2014/B Study. Blood.

[ref-308748] Short N., Kantarjian H., Ravandi F., Yilmaz M., Kadia T., Thompson P., Huang H., Konopleva M., Ferrajoli A., Jain N., Sasaki K., Alvarado Y., Borthakur G., Dinardo C., Ohanian M., Macaron W., Kornblau S., Zhao M., Kwari M., Loiselle C., Delumpa R., Milton A., Rivera J., Lewis S., Garris R., Jabbour E. (2022). Hyper-CVAD with sequential blinatumomab, with or without inotuzumab ozogamycin, in adults with newly diagnosed Philadelphia chromosome-negative B-cell acute lymphoblastic leukemia. Hemasphere.

[ref-308749] Litzow M., Sun Z., Paietta E., Mattison R. J. (2022). Consolidation Therapy with Blinatumomab Improves Overall Survival in Newly Diagnosed Adult Patients with B-Lineage Acute Lymphoblastic Leukemia in Measurable Residual Disease Negative Remission: Results from the ECOG-ACRIN E1910 Randomized Phase III National Cooperative Clinical Trials Network Trial. Blood.

[ref-308750] Stelljes M., Alakel N., Wäsch R., Scholl S. (2022). Inotuzumab Ozogamicin Induction Followed By Standard Chemotherapy Yields High Remission Rates and Promising Survival in Older (>55 Years) Patients with De Novo B-Lymphoblastic Leukemia (GMALL-Initial1 Trial). Blood.

[ref-308751] Chevallier P., Leguay T., Kim R., Delord M.. (2022). Fractionated Inotuzumab Ozogamicin Combined with Low-Intensity Chemotherapy in Older Patients with Newly Diagnosed CD22+ Philadelphia Chromosome (Ph)-Negative B-Cell Precursor (BCP) Acute Lymphoblastic Leukemia (ALL): Results of the EWALL-INO Study. Blood.

[ref-308752] Gökbuget N., Stoltefuß A., Topp M. S., Schwartz S.. (2021). Dose Reduced Chemotherapy in Sequence with Blinatumomab for Newly Diagnosed Older Patients with B-Precursor Adult Lymphoblastic Leukemia (ALL): Results of the Ongoing GMALL Bold Trial. Blood.

[ref-308753] Robak T., Szmigielska-Kapłon A., Wrzesień-Kuś A., Wierzbowska A., Skotnicki A. B., Piatkowska-Jakubas B., Kuliczkowski K., Mazur G., Zduńczyk A., Stella-Hołowiecka B., Hołowiecki J., Dwilewicz-Trojaczek J., Madry K., Dmoszyńska A., Cioch M. (2004). Acute lymphoblastic leukemia in elderly: the Polish Adult Leukemia Group (PALG) experience. Ann Hematol.

[ref-308754] Rowntree C. J., Kirkwood A. A., Clifton-Hadley L., Farah N., Mansour M. R.. (2021). First Analysis of the UKALL14 Randomized Trial to Determine Whether the Addition of Nelarabine to Standard Chemotherapy Improves Event Free Survival in Adults with T-Cell Acute Lymphoblastic Leukaemia (CRUK/09/006). Blood.

[ref-308755] Morita K., Jain N., Kantarjian H., akahashi K., Fang H., Konopleva M., El Hussein S., Wang F., Short N.J., Maiti A., Sasaki K., Garcia-Manero G., Konoplev S., Ravandi F., Khoury J.D., Jabbour E. (2021). Outcome of T-cell acute lymphoblastic leukemia/lymphoma: Focus on near-ETP phenotype and differential impact of nelarabine. Am J Hematol.

[ref-308756] Den Boer M. L., van Slegtenhorst M., De Menezes R. X., Cheok M. H., Buijs-Gladdines J. G., Peters S. T., Van Zutven L. J., Beverloo H. B., Van der Spek P. J., Escherich G., Horstmann M. A., Janka-Schaub G. E., Kamps W. A., Evans W. E., Pieters R. (2009). A subtype of childhood acute lymphoblastic leukaemia with poor treatment outcome: a genome-wide classification study. The Lancet Oncology.

[ref-308757] Roberts K. G. (2017). The biology of Philadelphia chromosome-like ALL. Best Pract Res Clin Haematol.

[ref-308758] Roberts K. G., Li Y., Payne-Turner D., Yang Y. L., Pei D., McCastlain K., Ding L., Lu C., Song G., Ma J., Becksfort J., Rusch M., Chen S. C., Easton J., Cheng J., Boggs K., Santiago-Morales N., Iacobucci I., Fulton R. S., Wen J., Valentine M., Cheng C., Paugh S. W., Devidas M., Chen I. M., Reshmi S., Smith A., Hedlund E., Gupta P., Nagahawatte P., Wu G., Chen X., Yergeau D., Vadodaria B., Mulder H., Winick N. J., Larsen E. C., Carroll W. L., Heerema N. A., Carroll A. J., Grayson G., Tasian S. K., Moore A. S., Keller F., Frei-Jones M., Whitlock J. A., Raetz E. A., White D. L., Hughes T. P., Guidry Auvil A., Smith M. A., Marcucci G., Bloomfield C. D., Mrózek K., Kohlschmidt J., Stock W., Kornblau S. M., Konopleva M., Paietta E., Pui C. H., Jeha S., Relling M. V., Evans W. E., Gerhard D. S., Gastier-Foster J. M., Mardis E., Wilson R. K., Loh M. L., Downing J. R., Hunger S. P., Willman C. L., Zhang J., Mullighan C. G. (2014). Targetable kinase-activating lesions in Ph-like acute lymphoblastic leukemia. New Engl J Med.

[ref-308759] Burmeister T., Schwartz S., Bartram C.R., Gökbuget N., Hoelzer D., Thiel E., GMALL study group (2008). Patients' age and BCR-ABL frequency in adult B-precursor ALL: a retrospective analysis from the GMALL study group. Blood.

[ref-308760] Moorman A. V., Harrison C. J., Buck G. A., Richards S. M., Secker-Walker L. M., Martineau M., Vance G. H., Cherry A. M., Higgins R. R., Fielding A. K., Foroni L., Paietta E., Tallman M. S., Litzow M. R., Wiernik P. H., Rowe J. M., Goldstone A. H., Dewald G. W., Adult Leukaemia Working Party, Medical Research Council/National Cancer Research Institute (2007). Karyotype is an independent prognostic factor in adult acute lymphoblastic leukemia (ALL): analysis of cytogenetic data from patients treated on the Medical Research Council (MRC) UKALLXII/Eastern Cooperative Oncology Group (ECOG) 2993 trial. Blood.

[ref-308761] Thomas D.A., Faderl S., Cortes J., O'Brien S., Giles F.J., Kornblau S.M., Garcia-Manero G., Keating M.J., Andreeff M., Jeha S., Beran M., Verstovsek S., Pierce S., Letvak L., Salvado A., Champlin R., Talpaz M., Kantarjian H. (2004). Treatment of Philadelphia chromosome-positive acute lymphocytic leukemia with hyper-CVAD and imatinib mesylate. Blood.

[ref-308762] de Labarthe A., Rousselot P., Huguet-Rigal F., Delabesse E., Witz F., Maury S., Réa D., Cayuela J. M., Vekemans M. C., Reman O., Buzyn A., Pigneux A., Escoffre M., Chalandon Y., MacIntyre E., Lhéritier V., Vernant J. P., Thomas X., Ifrah N., Dombret H., Group for Research on Adult Acute Lymphoblastic Leukemia (GRAALL) (2007). Imatinib combined with induction or consolidation chemotherapy in patients with de novo Philadelphia chromosome-positive acute lymphoblastic leukemia: results of the GRAAPH-2003 study. Blood.

[ref-308763] Wassmann B., Pfeifer H., Goekbuget N., Beelen D. W., Beck J., Stelljes M., Bornhäuser M., Reichle A., Perz J., Haas R., Ganser A., Schmid M., Kanz L., Lenz G., Kaufmann M., Binckebanck A., Brück P., Reutzel R., Gschaidmeier H., Schwartz S., Hoelzer D., Ottmann O. G. (2006). Alternating versus concurrent schedules of imatinib and chemotherapy as front-line therapy for Philadelphia-positive acute lymphoblastic leukemia (Ph+ ALL). Blood.

[ref-308764] Yanada M., Sugiura I., Takeuchi J., Akiyama H., Maruta A., Ueda Y., Usui N., Yagasaki F., Yujiri T., Takeuchi M., Nishii K., Kimura Y., Miyawaki S., Narimatsu H., Miyazaki Y., Ohtake S., Jinnai I., Matsuo K., Naoe T., Ohno R., Japan Adult Leukemia Study Group (2008). Prospective monitoring of BCR-ABL1 transcript levels in patients with Philadelphia chromosome-positive acute lymphoblastic leukaemia undergoing imatinib-combined chemotherapy. Br J Haematol.

[ref-308765] Ribera J. M., Oriol A., Gonzalez M., Vidriales B., Brunet S., Esteve J., Del Potro E., Rivas C., Moreno M. J., Tormo M., Martín-Reina V., Sarrá J., Parody R., de Oteyza J. P., Bureo E., Bernal M. T. (2010). Concurrent intensive chemotherapy and imatinib before and after stem cell transplantation in newly diagnosed Philadelphia chromosome-positive acute lymphoblastic leukemia. Final results of the CSTIBES02 trial. Haematologica.

[ref-308766] Mizuta S., Matsuo K., Yagasaki F., Yujiri T., Hatta Y., Kimura Y., Ueda Y., Kanamori H., Usui N., Akiyama H., Miyazaki Y., Ohtake S., Atsuta Y., Sakamaki H., Kawa K., Morishima Y., Ohnishi K., Naoe T., Ohno R. (2011). Pre-transplant imatinib-based therapy improves the outcome of allogeneic hematopoietic stem cell transplantation for BCR-ABL positive acute lymphoblastic leukemia. Leukemia.

[ref-308767] Chalandon Y., Thomas X., Hayette S., Cayuela J. M., Abbal C., Huguet F., Raffoux E., Leguay T., Rousselot P., Lepretre S., Escoffre-Barbe M., Maury S., Berthon C., Tavernier E., Lambert J. F., Lafage-Pochitaloff M., Lhéritier V., Chevret S., Ifrah N., Dombret H. (2015). Randomized study of reduced-intensity chemotherapy combined with imatinib in adults with Ph-positive acute lymphoblastic leukemia. Blood.

[ref-308768] Pfeifer H., Wassmann B., Bethge W., Dengler J., Bornhäuser M., Stadler M., Beelen D., Vucinic V., Burmeister T., Stelljes M., Faul C., Dreger P., Kiani A., Schäfer-Eckart K., Schwerdtfeger R., Lange E., Kubuschok B., Horst H.A., Gramatzki M., Brück P., Serve H., Hoelzer D., Gökbuget N., Ottmann O.G., GMALL Study Group (2013). Randomized comparison of prophylactic and minimal residual disease-triggered imatinib after allogeneic stem cell transplantation for BCR-ABL1-positive acute lymphoblastic leukemia. Leukemia.

[ref-308769] Giebel S., Czyz A., Ottmann O., Baron F., Brissot E., Ciceri F., Cornelissen J. J., Esteve J., Gorin N. C., Savani B., Schmid C., Mohty M., Nagler A. (2016). Use of tyrosine kinase inhibitors to prevent relapse after allogeneic hematopoietic stem cell transplantation for patients with Philadelphia chromosome-positive acute lymphoblastic leukemia: A position statement of the Acute Leukemia Working Party of the European Society for Blood and Marrow Transplantation. Cancer.

[ref-308770] Shen S., Chen X., Cai J., Yu J., Gao J., Hu S., Zhai X., Liang C., Ju X., Jiang H., Jin R., Wu X., Wang N., Tian X., Pan K., Jiang H., Sun L., Fang Y., Li C.K., Hu Q., Yang M., Zhu Y., Zhang H., Li C., Pei D., Jeha S., Yang J.J., Cheng C., Tang J., Zhu X., Pui C.H. (2020). Effect of Dasatinib vs Imatinib in the Treatment of Pediatric Philadelphia Chromosome-Positive Acute Lymphoblastic Leukemia: A Randomized Clinical Trial. JAMA Oncol.

[ref-308771] Ravandi F., O'Brien S. M., Cortes J. E., Thomas D. M., Garris R., Faderl S., Burger J. A., Rytting M. E., Ferrajoli A., Wierda W. G., Verstovsek S., Champlin R., Kebriaei P., McCue D. A., Huang X., Jabbour E., Garcia-Manero G., Estrov Z., Kantarjian H. M. (2015). Long-term follow-up of a phase 2 study of chemotherapy plus dasatinib for the initial treatment of patients with Philadelphia chromosome-positive acute lymphoblastic leukemia. Cancer.

[ref-308772] Jabbour E., DerSarkissian M., Duh M. S., McCormick N., Cheng W. Y., McGarry L. J., Souroutzidis A., Huang H., O'Brien S., Ravandi F., Kantarjian H. M. (2018). Efficacy of Ponatinib Versus Earlier Generation Tyrosine Kinase Inhibitors for Front-line Treatment of Newly Diagnosed Philadelphia-positive Acute Lymphoblastic Leukemia. Clin Lymphoma Myeloma Leuk.

[ref-308773] Rousselot P., Coude M. M., Gokbuget N., Gambacorti Passerini C., Hayette S., Cayuela J. M., Huguet F., Leguay T., Chevallier P., Salanoubat C., Bonmati C., Alexis M., Hunault M., Glaisner S., Agape P., Berthou C., Jourdan E., Fernandes J., Sutton L., Banos A., Reman O., Lioure B., Thomas X., Ifrah N., Lafage-Pochitaloff M., Bornand A., Morisset L., Robin V., Pfeifer H., Delannoy A., Ribera J., Bassan R., Delord M., Hoelzer D., Dombret H., Ottmann O. G., European Working Group on Adult ALL (EWALL) group (2016). Dasatinib and low-intensity chemotherapy in elderly patients with Philadelphia chromosome-positive ALL. Blood.

[ref-308774] Foà R., Bassan R., Vitale A., Elia L., Piciocchi A., Puzzolo M.C., Canichella M., Viero P., Ferrara F., Lunghi M., Fabbiano F., Bonifacio M., Fracchiolla N., Di Bartolomeo P., Mancino A., De Propris M.S., Vignetti M., Guarini A., Rambaldi A., Chiaretti S., GIMEMA Investigators (2020). Dasatinib-Blinatumomab for Ph-Positive Acute Lymphoblastic Leukemia in Adults. N Engl J Med.

[ref-308775] Jabbour E., Short N. J., Jain N., Huang X., Montalban-Bravo G., Banerjee P., Rezvani K., Jiang X., Kim K. H., Kanagal-Shamanna R., Khoury J. D., Patel K., Kadia T. M., Daver N., Chien K., Alvarado Y., Garcia-Manero G., Issa G. C., Haddad F. G., Kwari M., Thankachan J., Delumpa R., Macaron W., Garris R., Konopleva M., Ravandi F., Kantarjian H. (2023). Ponatinib and blinatumomab for Philadelphia chromosome-positive acute lymphoblastic leukaemia: a US, single-centre, single-arm, phase 2 trial. Lancet Haematol.

[ref-308776] Shem-Tov N., Peczynski C., Labopin M., Itälä-Remes M., Blaise D., Labussière-Wallet H., Socié G., Kröger N., Mielke S., Afanasyev B., Chevallier P., Tischer J., Helbig G., Jindra P., Peric Z., Giebel S., Mohty M., Nagler A. (2020). Haploidentical vs. unrelated allogeneic stem cell transplantation for acute lymphoblastic leukemia in first complete remission: on behalf of the ALWP of the EBMT. Leukemia.

[ref-308777] Nagler A., Labopin M., Houhou M., Aljurf M., Mousavi A., Hamladji R. M., Al Zahrani M., Bondarenko S., Arat M., Angelucci E., Koc Y., Gülbas Z., Sica S., Bourhis J. H., Canaani J., Brissot E., Giebel S., Mohty M. (2021). Outcome of haploidentical versus matched sibling donors in hematopoietic stem cell transplantation for adult patients with acute lymphoblastic leukemia: a study from the Acute Leukemia Working Party of the European Society for Blood and Marrow Transplantation. J Hematol Oncol.

[ref-308778] Nagler A., Kanate A. S., Labopin M., Ciceri F., Angelucci E., Koc Y., Gülbas Z., Arcese W., Tischer J., Pioltelli P., Ozdogu H., Afanasyev B., Wu D., Arat M., Peric Z., Giebel S., Savani B., Mohty M. (2021). Post-transplant cyclophosphamide versus anti-thymocyte globulin for graft-versus-host disease prevention in haploidentical transplantation for adult acute lymphoblastic leukemia. Haematologica.

[ref-308779] Nagler A., Dholaria B., Labopin M., Savani B. N., Angelucci E., Koc Y., Arat M., Pioltelli P., Sica S., Gülbas Z., Tischer J., Bernasconi P., Pavlu J., Socié G., Blaise D., Rigacci L., Martino M., Diez-Martin J. L., Perić Z., Giebel S., Mohty M. (2020). Bone marrow versus mobilized peripheral blood stem cell graft in T-cell-replete haploidentical transplantation in acute lymphoblastic leukemia. Leukemia.

[ref-308780] Nagler A., Labopin M., Koc Y., Angelucci E., Tischer J., Arat M., Pioltelli P., Bernasconi P., Chiusolo P., Diez-Martin J. L., Sanz J., Ciceri F., Peric Z., Giebel S., Canaani J., Mohty M. (2021). Outcome of T-cell-replete haploidentical stem cell transplantation improves with time in adults with acute lymphoblastic leukemia: A study from the Acute Leukemia Working Party of the European Society for Blood and Marrow Transplantation. Cancer.

[ref-308781] Pavlů J., Labopin M., Niittyvuopio R., Socié G., Yakoub-Agha I., Wu D., Remenyi P., Passweg J., Beelen D.W., Aljurf M., Kröger N., Labussière-Wallet H., Perić Z., Giebel S., Nagler A., Mohty M. (2019). Measurable residual disease at myeloablative allogeneic transplantation in adults with acute lymphoblastic leukemia: a retrospective registry study on 2780 patients from the acute leukemia working party of the EBMT. J Hematol Oncol.

[ref-308782] Cahu X., Labopin M., Giebel S., Aljurf M., Kyrcz-Krzemien S., Socié G., Eder M., Bonifazi F., Bunjes D., Vigouroux S., Michallet M., Stelljes M., Zuckerman T., Finke J., Passweg J., Yakoub-Agha I., Niederwieser D., Sucak G., Sengeløv H., Polge E., Nagler A., Esteve J., Mohty M., Acute Leukemia Working Party of EBMT (2016). Impact of conditioning with TBI in adult patients with T-cell ALL who receive a myeloablative allogeneic stem cell transplantation: a report from the acute leukemia working party of EBMT. Bone Marrow Transplant.

[ref-308783] Peters C., Dalle J. H., Locatelli F., Poetschger U., Sedlacek P., Buechner J., Shaw P. J., Staciuk R., Ifversen M., Pichler H., Vettenranta K., Svec P., Aleinikova O., Stein J., Güngör T., Toporski J., Truong T. H., Diaz-de-Heredia C., Bierings M., Ariffin H., Essa M., Burkhardt B., Schultz K., Meisel R., Lankester A., Ansari M., Schrappe M., von Stackelberg A., Balduzzi A., Corbacioglu S., Bader P. (2021). Total Body Irradiation or Chemotherapy Conditioning in Childhood ALL: A Multinational, Randomized, Noninferiority Phase III Study. J Clin Oncol.

[ref-308784] Zhang H., Fan Z., Huang F., Han L., Xu Y., Xu N., Deng L., Wang S., Lin D., Luo X., Zhang Q., Liu X., Li X., Liang X., Xie S., Qu H., Yu S., Zhou H., Shi P., Xuan L., Lin R., Liu H., Jin H., Sun J., Liu Q. (2023). Busulfan Plus Cyclophosphamide Versus Total Body Irradiation Plus Cyclophosphamide for Adults Acute B Lymphoblastic Leukemia: An Open-Label, Multicenter, Phase III Trial. J Clin Oncol.

[ref-308785] Czerw T., Labopin M., Giebel S., Socié G., Volin L., Fegueux N., Masszi T., Blaise D., Chaganti S., Cornelissen J. J., Passweg J., Maertens J., Itälä-Remes M., Wu D., Mohty M., Nagler A. (2018). Anti-thymocyte globulin improves survival free from relapse and graft-versus-host disease after allogeneic peripheral blood stem cell transplantation in patients with Philadelphia-negative acute lymphoblastic leukemia: An analysis by the Acute Leukemia Working Party of the EBMT. Cancer.

[ref-308786] Giebel S., Labopin M., Czerw T., Socié G., Blaise D., Ghavamzadeh A., Passweg J., Ljungman P., Poiré X., Chevallier P., Reményi P., Rambaldi A., Anafasyev B., Fegueux N., Rovira M., Itälä-Remes M., Bornhäuser M., Mohty M., Nagler A. (2019). Impact of anti-thymocyte globulin on results of allogeneic peripheral blood stem cell transplantation for patients with Philadelphia-positive acute lymphoblastic leukaemia: An analysis by the Acute Leukemia Working Party of the EBMT. Eur J Cancer.

[ref-308787] Maude S.L., Laetsch T.W., Buechner J., Rives S., Boyer M., Bittencourt H., Bader P., Verneris M.R., Stefanski H.E., Myers G.D., Qayed M., De Moerloose B., Hiramatsu H., Schlis K., Davis K.L., Martin P.L., Nemecek E.R., Yanik G.A., Peters C., Baruchel A., Boissel N., Mechinaud F., Balduzzi A., Krueger J., June C.H., Levine B.L., Wood P., Taran T., Leung M., Mueller K.T., Zhang Y., Sen K., Lebwohl D., Pulsipher M.A., Grupp S.A. (2018). Tisagenlecleucel in Children and Young Adults with B-Cell Lymphoblastic Leukemia. N Engl J Med.

[ref-308788] Shah B. D., Ghobadi A., Oluwole O. O., Logan A. C., Boissel N., Cassaday R. D., Leguay T., Bishop M. R., Topp M. S., Tzachanis D., O'Dwyer K. M., Arellano M. L., Lin Y., Baer M. R., Schiller G. J., Park J. H., Subklewe M., Abedi M., Minnema M. C., Wierda W. G., DeAngelo D. J., Stiff P., Jeyakumar D., Feng C., Dong J., Shen T., Milletti F., Rossi J. M., Vezan R., Masouleh B. K., Houot R. (2021). KTE-X19 for relapsed or refractory adult B-cell acute lymphoblastic leukaemia: phase 2 results of the single-arm, open-label, multicentre ZUMA-3 study. Lancet.

[ref-308789] Tomasik J., Jasiński M., Basak G. W. (2022). Next generations of CAR-T cells - new therapeutic opportunities in hematology?. Front Immunol.

[ref-308790] Yang Y., Bi X., Gergis M., Yi D., Hsu J., Gergis U. (2022). Allogeneic Chimeric Antigen Receptor T Cells for Hematologic Malignancies. Hematol Oncol Stem Cell Ther.

